# Bis(1,2,3,4-tetra­hydro­quinolin-6-yl)methane

**DOI:** 10.1107/S1600536808025464

**Published:** 2008-08-13

**Authors:** Li Shen, Qi Liu, Hai-Jun Shen

**Affiliations:** a Medical College, Yangzhou University, Yangzhou 225001, Jiangsu Province, People’s Republic of China

## Abstract

The asymmetric unit of the title compound, C_19_H_22_N_2_, contains one half-mol­ecule. The 1,2,3,4-tetra­hydro­quinoline units are linked by a methyl­ene bridge, which lies on a twofold rotation axis. The non-aromatic ring adopts a flattened-boat conformation. The dihedral angle between the two symmetry-related benzene rings is 64.03 (7)°.

## Related literature

For general background, see: Xiao *et al.* (2008*a* ▶,*b* ▶,*c* ▶); Xiao *et al.* (2007*a* ▶,*b* ▶); Xue *et al.* (2007 ▶). For ring conformation puckering parameters, see: Cremer & Pople (1975 ▶).
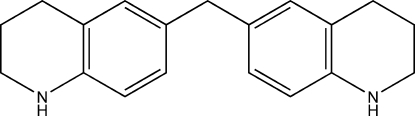


## Experimental

### 

#### Crystal data

C_19_H_22_N_2_
*M* *_r_* = 278.39
Orthorhombic, 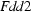

*a* = 17.515 (3) Å
*b* = 29.660 (4) Å
*c* = 5.7678 (8) Å
*V* = 2996.2 (8) Å^3^
*Z* = 8
Mo *K*α radiation
μ = 0.07 mm^−1^
*T* = 292 (2) K
0.30 × 0.20 × 0.20 mm

#### Data collection

Enraf–Nonius CAD-4 diffractometer
Absorption correction: ψ scan (North *et al.*, 1968 ▶) *T*_min_ = 0.979, *T*_max_ = 0.986
4545 measured reflections
814 independent reflections
773 reflections with *I* > 2σ(*I*)
*R*_int_ = 0.073

#### Refinement

*R*[*F* ^2^ > 2σ(*F* ^2^)] = 0.048
*wR*(*F* ^2^) = 0.138
*S* = 1.07
814 reflections
100 parameters
2 restraints
H atoms treated by a mixture of independent and constrained refinement
Δρ_max_ = 0.28 e Å^−3^
Δρ_min_ = −0.21 e Å^−3^

### 

Data collection: *CAD-4 Software* (Enraf–Nonius, 1989 ▶); cell refinement: *CAD-4 Software*; data reduction: *XCAD4* (Harms & Wocadlo, 1995 ▶); program(s) used to solve structure: *SHELXS97* (Sheldrick, 2008 ▶); program(s) used to refine structure: *SHELXL97* (Sheldrick, 2008 ▶); molecular graphics: *PLATON* (Spek, 2003 ▶); software used to prepare material for publication: *SHELXTL* (Sheldrick, 2008 ▶).

## Supplementary Material

Crystal structure: contains datablocks global, I. DOI: 10.1107/S1600536808025464/hk2506sup1.cif

Structure factors: contains datablocks I. DOI: 10.1107/S1600536808025464/hk2506Isup2.hkl

Additional supplementary materials:  crystallographic information; 3D view; checkCIF report

## Figures and Tables

**Fig. 1.**
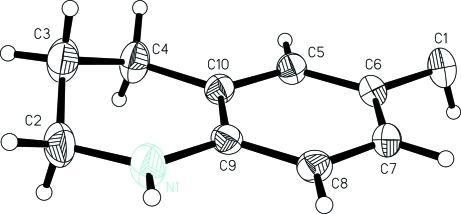
The molecular structure of the title molecule, with the atom-numbering scheme [symmetry code: (a) -x, 1 - y, z].

## supplementary crystallographic information

### Comment

Nitrogen heterocyclic compounds show diverse biological activities such as antiproliferative (Xiao *et al.*,  2008*a*,b), antibacterial (Xiao *et al.*,  2007*a*; Xue *et al.*,  2007; Xiao *et al.*,  2008*c*), and urease inhibitory (Xiao *et al.*,  2007*b*) activities. The title compound is a heterocyclic compound, which may be used for screening the biological activities. We report herein its crystal structure.

The asymmetric unit of the title compound (Fig. 1) contains one-half molecule. 1,2,3,4-Tetrahydroquinoline moieties are joined by a methylene bridge. Ring A (N1/C2-C4/C9/C10) adopts flattened-boat [φ = 126.28 (2)°, θ = 26.77 (3)°] conformation, having total puckering amplitude, Q_T_, of 0.448 (3) Å (Cremer & Pople, 1975). The dihedral angle between the two symmetry related phenyl rings is 64.03 (7)°.

### Experimental

Heating of 4,4'-methylenedibenzenamine (2 g), bis(4-nitrophenyl)methane (0.5 g), H_3_AsO_4_ (1.5 g), concentrated H_2_SO_4_ (3 ml), and glycerol (8.6 ml) at 413 K for 5 h, addition of water, removal of resinous matter, making alkalization with NaOH, taking up in ether, dehydration with K_2_CO_3_, and recrystallization of the residue from alcohol gives diquinolin-6-ylmethane. The resulting product (1 g) was subsequently heated with Sn (5.5 g) and HCl (22 ml, 32%) in a water bath for 8 h, addition of water, precipitation of the Sn as Sn(OH)_2_ by NaOH, taking up in ether, and drying with K_2_CO_3_, gives the title compound (yield; 0.9 g), which was recrystallized from petroleum ether-ethyl acetate to give colorless prisms.

### Refinement

H1 atom (for bridging CH_2_) was located in difference syntheses and refined isotropically [C-H = 0.97 (2) Å and U_iso_(H) = 0.067 (10) Å^2^]. The remaining H atoms were positioned geometrically, with N-H = 0.86 Å (for NH) and C-H = 0.93 and 0.97 Å for aromatic and methylene H, respectively, and constrained to ride on their parent atoms with U_iso_(H) = 1.2U_eq_(C).

### Crystal data

**Table:**

C_19_H_22_N_2_	*F*_000_ = 1200
*M**_r_* = 278.39	*D*_x_ = 1.234 Mg m^−^^3^
Orthorhombic, *F**d**d*2	Mo *K*α radiation λ = 0.71073 Å
Hall symbol: F 2 -2d	Cell parameters from 1297 reflections
*a* = 17.515 (3) Å	θ = 2.9–25.2º
*b* = 29.660 (4) Å	µ = 0.07 mm^−^^1^
*c* = 5.7678 (8) Å	*T* = 292 (2) K
*V* = 2996.2 (8) Å^3^	Prism, colorless
*Z* = 8	0.30 × 0.20 × 0.20 mm

### Data collection

**Table:**

Enraf–Nonius CAD-4 diffractometer	814 independent reflections
Radiation source: fine-focus sealed tube	773 reflections with *I* > 2σ(*I*)
Monochromator: graphite	*R*_int_ = 0.073
*T* = 292(2) K	θ_max_ = 26.0º
ω/2θ scans	θ_min_ = 2.7º
Absorption correction: ψ scan(North *et al.*, 1968)	*h* = −21→21
*T*_min_ = 0.979, *T*_max_ = 0.986	*k* = −36→32
4545 measured reflections	*l* = −7→6

### Refinement

**Table:**

Refinement on *F*^2^	Secondary atom site location: difference Fourier map
Least-squares matrix: full	Hydrogen site location: inferred from neighbouring sites
*R*[*F*^2^ > 2σ(*F*^2^)] = 0.048	H atoms treated by a mixture of independent and constrained refinement
*wR*(*F*^2^) = 0.138	*w* = 1/[σ^2^(*F*_o_^2^) + (0.0996*P*)^2^ + 0.9957*P*] where *P* = (*F*_o_^2^ + 2*F*_c_^2^)/3
*S* = 1.08	(Δ/σ)_max_ < 0.001
814 reflections	Δρ_max_ = 0.28 e Å^−^^3^
100 parameters	Δρ_min_ = −0.21 e Å^−^^3^
2 restraints	Extinction correction: none
Primary atom site location: structure-invariant direct methods	

### Special details

**Table:**

Geometry. All esds (except the esd in the dihedral angle between two l.s. planes) are estimated using the full covariance matrix. The cell esds are taken into account individually in the estimation of esds in distances, angles and torsion angles; correlations between esds in cell parameters are only used when they are defined by crystal symmetry. An approximate (isotropic) treatment of cell esds is used for estimating esds involving l.s. planes.
Refinement. Refinement of F^2^ against ALL reflections. The weighted R-factor wR and goodness of fit S are based on F^2^, conventional R-factors R are based on F, with F set to zero for negative F^2^. The threshold expression of F^2^ > 2sigma(F^2^) is used only for calculating R-factors(gt) etc. and is not relevant to the choice of reflections for refinement. R-factors based on F^2^ are statistically about twice as large as those based on F, and R- factors based on ALL data will be even larger.

### Fractional atomic coordinates and isotropic or equivalent isotropic displacement parameters (Å^2^)

**Table:**

	*x*	*y*	*z*	*U*_iso_*/*U*_eq_	
N1	0.26094 (12)	0.44383 (8)	1.0848 (5)	0.0605 (7)	
H11A	0.2700	0.4566	1.2159	0.073*	
C1	0.0000	0.5000	0.5624 (6)	0.0519 (9)	
H1	0.0128 (16)	0.5254 (7)	0.464 (5)	0.067 (10)*	
C2	0.31123 (16)	0.40914 (9)	1.0048 (7)	0.0637 (9)	
H2A	0.3360	0.3951	1.1368	0.076*	
H2B	0.3505	0.4224	0.9080	0.076*	
C3	0.27005 (17)	0.37452 (10)	0.8715 (8)	0.0662 (9)	
H3A	0.2357	0.3584	0.9740	0.079*	
H3B	0.3064	0.3530	0.8094	0.079*	
C4	0.22470 (16)	0.39493 (9)	0.6743 (6)	0.0579 (8)	
H4A	0.2595	0.4045	0.5530	0.070*	
H4B	0.1911	0.3722	0.6097	0.070*	
C5	0.11375 (13)	0.44942 (7)	0.6314 (5)	0.0415 (6)	
H5	0.1004	0.4345	0.4954	0.050*	
C6	0.06877 (12)	0.48523 (8)	0.7038 (5)	0.0407 (6)	
C7	0.09023 (12)	0.50718 (8)	0.9058 (5)	0.0434 (6)	
H7	0.0615	0.5315	0.9581	0.052*	
C8	0.15365 (13)	0.49360 (8)	1.0313 (5)	0.0435 (6)	
H8	0.1667	0.5088	1.1667	0.052*	
C9	0.19817 (12)	0.45735 (7)	0.9568 (5)	0.0384 (5)	
C10	0.17797 (12)	0.43464 (7)	0.7524 (5)	0.0390 (6)	

### Atomic displacement parameters (Å^2^)

**Table:**

	*U*^11^	*U*^22^	*U*^33^	*U*^12^	*U*^13^	*U*^23^
N1	0.0578 (13)	0.0610 (13)	0.0628 (17)	0.0115 (10)	−0.0273 (12)	−0.0153 (13)
C1	0.0451 (19)	0.068 (2)	0.042 (2)	0.0156 (17)	0.000	0.000
C2	0.0537 (15)	0.0635 (16)	0.074 (2)	0.0170 (11)	−0.0201 (16)	0.0025 (16)
C3	0.0623 (16)	0.0582 (15)	0.078 (2)	0.0198 (12)	−0.0097 (17)	−0.0042 (16)
C4	0.0622 (16)	0.0558 (14)	0.0558 (18)	0.0202 (12)	−0.0111 (15)	−0.0126 (14)
C5	0.0420 (12)	0.0442 (11)	0.0384 (13)	0.0016 (9)	−0.0014 (11)	−0.0069 (10)
C6	0.0324 (10)	0.0460 (10)	0.0437 (13)	0.0024 (8)	0.0016 (10)	0.0000 (11)
C7	0.0383 (11)	0.0416 (11)	0.0504 (15)	0.0045 (9)	0.0061 (10)	−0.0052 (11)
C8	0.0440 (12)	0.0432 (11)	0.0433 (14)	−0.0040 (9)	−0.0018 (11)	−0.0113 (11)
C9	0.0345 (11)	0.0392 (10)	0.0416 (13)	−0.0032 (8)	−0.0034 (10)	0.0013 (10)
C10	0.0398 (11)	0.0375 (10)	0.0398 (12)	0.0008 (8)	−0.0007 (10)	−0.0036 (11)

### Geometric parameters (Å, °)

**Table:**

N1—C2	1.431 (3)	C4—H4B	0.9700
N1—H11A	0.8600	C5—C6	1.387 (3)
C1—C6	1.519 (3)	C5—C10	1.394 (3)
C1—C6^i^	1.519 (3)	C5—H5	0.9300
C1—H1	0.97 (2)	C7—C6	1.386 (4)
C2—C3	1.472 (4)	C7—H7	0.9300
C2—H2A	0.9700	C8—C7	1.386 (3)
C2—H2B	0.9700	C8—H8	0.9300
C3—H3A	0.9700	C9—N1	1.384 (3)
C3—H3B	0.9700	C9—C8	1.396 (3)
C4—C3	1.514 (4)	C9—C10	1.403 (4)
C4—H4A	0.9700	C10—C4	1.503 (3)
			
C9—N1—C2	121.7 (3)	C10—C4—H4B	109.2
C9—N1—H11A	119.2	C3—C4—H4B	109.2
C2—N1—H11A	119.2	H4A—C4—H4B	107.9
C6—C1—C6^i^	115.1 (3)	C6—C5—C10	123.3 (2)
C6—C1—H1	110.8 (18)	C6—C5—H5	118.4
C6^i^—C1—H1	105.9 (19)	C10—C5—H5	118.4
N1—C2—C3	111.6 (2)	C7—C6—C5	117.3 (2)
N1—C2—H2A	109.3	C7—C6—C1	122.0 (2)
C3—C2—H2A	109.3	C5—C6—C1	120.6 (2)
N1—C2—H2B	109.3	C8—C7—C6	121.3 (2)
C3—C2—H2B	109.3	C8—C7—H7	119.3
H2A—C2—H2B	108.0	C6—C7—H7	119.3
C2—C3—C4	111.8 (2)	C7—C8—C9	120.7 (2)
C2—C3—H3A	109.3	C7—C8—H8	119.6
C4—C3—H3A	109.3	C9—C8—H8	119.6
C2—C3—H3B	109.3	N1—C9—C8	120.2 (2)
C4—C3—H3B	109.3	N1—C9—C10	120.6 (2)
H3A—C3—H3B	107.9	C8—C9—C10	119.2 (2)
C10—C4—C3	112.0 (2)	C5—C10—C9	118.2 (2)
C10—C4—H4A	109.2	C5—C10—C4	122.4 (2)
C3—C4—H4A	109.2	C9—C10—C4	119.4 (2)
			
C9—N1—C2—C3	−33.5 (4)	C9—C8—C7—C6	0.3 (4)
C6^i^—C1—C6—C7	−39.05 (19)	C8—C9—N1—C2	−175.1 (2)
C6^i^—C1—C6—C5	142.3 (3)	C10—C9—N1—C2	5.6 (4)
N1—C2—C3—C4	54.2 (4)	N1—C9—C8—C7	−179.4 (2)
C10—C4—C3—C2	−48.1 (4)	C10—C9—C8—C7	0.0 (4)
C10—C5—C6—C7	0.6 (4)	N1—C9—C10—C5	179.4 (2)
C10—C5—C6—C1	179.3 (2)	C8—C9—C10—C5	0.0 (3)
C6—C5—C10—C9	−0.3 (4)	N1—C9—C10—C4	0.7 (4)
C6—C5—C10—C4	178.3 (2)	C8—C9—C10—C4	−178.6 (2)
C8—C7—C6—C5	−0.6 (4)	C5—C10—C4—C3	−157.7 (2)
C8—C7—C6—C1	−179.2 (2)	C9—C10—C4—C3	20.9 (4)

Symmetry codes: (i) −*x*, −*y*+1, *z*.
